# USP39 interacts with SIRT7 to promote cervical squamous cell carcinoma by modulating autophagy and oxidative stress via FOXM1

**DOI:** 10.1186/s12967-023-04623-4

**Published:** 2023-11-13

**Authors:** Juanpeng Yu, Shuai Yuan, Jinglin Song, Shengsheng Yu

**Affiliations:** 1 Department of Gynecology, The Affiliated Huai’an No.1 People’s Hospital of Nanjing Medical University, No. 1 Huanghe West Road, Huaiyin District, Huai’an, 223300 Jiangsu China; 2 Department of Obstetrics and Gynecology, Langao County Hospital of Traditional Chinese Medicine, Ankang, 725400 Shaanxi China

**Keywords:** Cervical squamous cell carcinoma, SIRT7, USP39, Autophagy, Oxidative stress

## Abstract

**Background:**

Sirtuin 7 (SIRT7) is an oncogene that promotes tumor progression in various malignancies, however, its role and regulatory mechanism in cervical squamous cell carcinoma (CSCC) is unknown. Herein, we attempted to investigate the functional role and molecular mechanism of SIRT7 underlying CSCC progression.

**Methods:**

SIRT7 expression was evaluated in CSCC cells using various assays. We then used a series of function gain-and-loss experiments to determine the role of SIRT7 in CSCC progression. Furthermore, mechanism experiments were conducted to assess the interaction between SIRT7/USP39/FOXM1 in CSCC cells. Additionally, rescue assays were conducted to explore the regulatory function of USP39/FOXM1 in CSCC cellular processes.

**Results:**

SIRT7 was highly expressed in CSCC patient tissues and cell lines. SIRT7 deficiency showed significant repression on the proliferation, and autophagy of CSCC cells in vitro and tumorigenesis in vivo. Similarly, apoptosis and ROS production in CSCC cells were accelerated after the SIRT7 knockdown. Moreover, SIRT7 and USP39 were found colocalized in the cell nucleus. Interestingly, SIRT7 was revealed to deacetylate USP39 to promote its protein stability in CSCC cells. USP39 protein was also verified to be upregulated in CSCC tissues and cells. USP39 silencing showed suppressive effects on CSCC cell growth. Mechanistically, USP39 was revealed to upregulate SIRT7 by promoting the transcriptional activity of FOXM1. Rescue assays also indicated that SIRT7 promoted autophagy and inhibited ROS production in CSCC cells by regulating USP39/FOXM1.

**Conclusion:**

The SIRT7/USP39/FOXM1 positive feedback network regulates autophagy and oxidative stress in CSCC, thus providing a new direction for CSCC-targeted therapy.

**Graphical Abstract:**

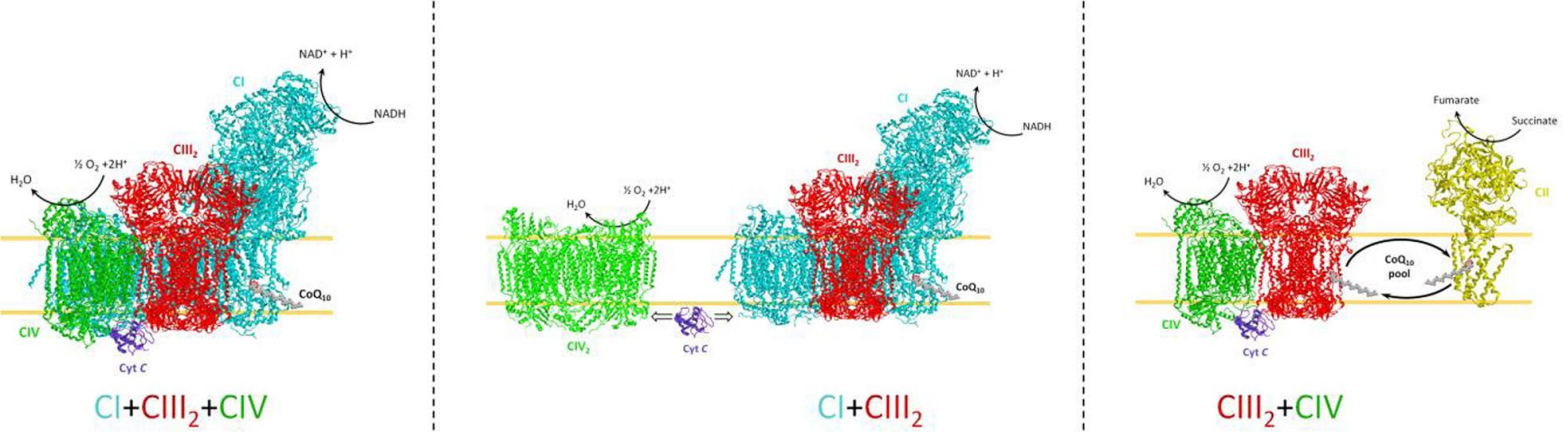

**Supplementary Information:**

The online version contains supplementary material available at 10.1186/s12967-023-04623-4.

## Introduction

Cervical cancer is the fourth in female cancer incidence and mortality, with approximately over 60,000 new cases and nearly 342,000 death cases globally [1]. Squamous cell carcinomas (SCC) and adenocarcinomas (ADC) are the most prevalent histological types of cervical cancer and account for around 70% and 25% of all cervical cancers [2, 3]. Factors such as sexually transmittable infections, smoking, multiple childbirths, and long-term use of oral contraceptives contribute to cervical cancer progression [4, 5]. The incidence of cervical cancer has declined in the past few decades due to the high-risk human papillomavirus (HPV) DNA testing and HPV vaccination. In spite of the advances, the 5 year survival rate in advanced cervical cancer patients is merely 16.7% [6]. This calls for the exploration of novel therapeutic strategies to improve the treatment outcome of cervical cancer patients.

Sirtuin 7 (SIRT7) is a member of the sirtuin family (SIRT1-7) of mammalian NAD^+^-dependent deacetylases intrinsically related to cellular metabolism [7]. Localized in the nucleus, SIRT7 is widely expressed in a variety of tissues and functions to deacetylate histones to regulate gene expression epigenetically [8]. Previous studies have also demonstrated that SIRT7 acts as an oncogene or anti-cancer gene in the progression of diverse cancers, regulating cell proliferation and autophagy, and serves as a stress regulator in physiological homeostasis [9–11]. For example, SIRT7 promotes the proliferation and androgen-induced autophagy in prostate cancer via the AR signaling [12]. SIRT7 knockdown is also reported to enhance the sensitivity of non-small cell lung cancer cells to gemcitabine by suppressing autophagy [13]. SIRT7 facilitates cell proliferation abilities and enhances cell invasiveness in colorectal cancer via the Raf-MEK-ERK signaling [14]. Whereas the function of SIRT7 in cervical squamous cell carcinomas is largely unknown.

Increasing attention has been paid to deubiquitinating enzymes (DUBs) for their role in genomic stability and tumor progression [15, 16]. They are suggested to participate in the regulation of autophagy and oxidative stress [17, 18]. Ubiquitin specific peptidase 39 (USP39) is a SR-associated protein (65 kDa) essentially engaged in RNA splicing. It is also reported as a DUB with no protease activity and can regulate downstream gene expression by modulating pre-mRNA splicing [19, 20]. Substantial literature has revealed that USP39 exerts oncogenic impact on the development of various malignancies such as osteosarcoma, hepatocellular carcinoma, and glioma [21–23]. Moreover, SIRT7 has been demonstrated to deacetylate USP39, increasing the stability, and promoting the oncogenic activity of USP39 in hepatocellular carcinoma development [24]. However, the relationship between SIRT7 and USP39 in cervical cancer is not known.

In this study, we aimed to elucidate the biological functions and the underlying mechanism of SIRT7 in cervical squamous cell carcinoma (CSCC) progression. We hypothesized that SIRT7 may regulate the autophagy and oxidative stress in cervical cancer through USP39. The findings of this work might offer novel targeted strategy for cervical cancer therapy.

## Materials and methods

### Patients tissue sample collection

The tumor (n = 40) and adjacent normal tissue samples (3 cm away from tumor, n = 40) were collected from patients diagnosed with CSCC by histopathological fine needle biopsy in our hospital. The tissues were identified by histopathological examinations and kept in liquid nitrogen. The study was approved by the Ethics Committee of The Affiliated Huai’an No.1 People’s Hospital of Nanjing Medical University. All participants agreed and signed the informed consent prior to our study.

### Cell culture and treatment

Human cervical squamous cell carcinoma cells (CaSki, SiHa, C-33A) and human normal cervical epithelial cells (Ect1/E6E7) were provided by the American Type Culture Collection (ATCC, Rockville, Md., USA). CaSKi cells are classified as epidermoid carcinoma and are reported to contain an integrated human papillomavirus type 16 genome (HPV-16, about 600 copies per cell) as well as sequences related to HPV-18. SiHa cells are classified as squamous cell carcinoma and are reported to be a hypertriploid human cell line with the modal chromosome number of 71, occurring in 24% of cells. C-33A cells are classified as epidermoid carcinoma and are reported to be a pseudodiploid human cell line with the modal chromosome number of 46, occurring in 70% of cells examined. CaSki cells were incubated in Roswell Park Memorial Institute-1640 medium (Thermo Fisher) with 10% Fetal Bovine Serum (FBS, Thermo Fisher), 100 units/mL penicillin, 100 µg/mL streptomycin. SiHa and C-33A cells were incubated in Minimum Essential Medium (MEM, Thermo Fisher) supplemented with 10% FBS. Ect1/E6E7 cells were incubated in Keratinocyte-Serum Free medium (Thermo Fisher). All cells were cultured in a humidified incubator at 37 °C and 5% CO_2_. For cycloheximide (CHX) treatment, 30 μM of CHX was added into cell culture medium followed by incubation for indicated time periods (0, 2, 4, 6, 8 h). Then proteins were extracted and subjected to western blot analysis.

### Cell transfection

Short hairpin RNAs targeted SIRT7 (shSIRT7#1: CCGGTCCACGGGAACATGTACATTGCTCGAGCAATGTACATGTTCCCGTGGATTTTTG, shUSP39#2: CCGGGTCCAGCCTGAAGGTTCTAAACTCGAGTTTAGAACCTTCAGGCTGGACTTTTTG) and USP39 (shUSP39#1: CCGGGCCGGGTATTGTGGGACTGAACTCGAGTTCAGTCCCACAATACCCGGCTTTTT, shUSP39#2: CCGGGATTTGGAAGAGGCGAGATAACTCGAGTTATCTCGCCTCTTCCAAATCTTTTTG) and negative control (shNC) were constructed by Invitrogen. For FOXM1 and USP39 overexpression, pcDNA3.1/FOXM1 and pcDNA3.1/USP39 (Invitrogen) vectors were used while pcDNA3.1 with empty pcDNA3.1 vector was used as the negative control. When cells confluence reached 80%, the above vectors and plasmids were transfected into CaSki and SiHa cells with lipofectamine 2000 (11668019, Invitrogen). After 48 h, cells were collected for following analyses.

### qRT-PCR

A FastPure Cell/Tissue Total RNA Isolation Mini Kit (RC101-01, Vazyme, Nanjing, China) was used to collect total RNA from tissues and cells, and a HiScript II 1st Strand cDNA Synthesis Kit (R211-01, Vazyme, China) was used for RNA reverse transcription. qRT-PCR was conducted with a SYBR Green PCR Master Mix (A46012, Thermo Fisher). Relative expression of SIRT7, USP39 and FOXM1 was analyzed using the 2^−ΔΔCt^ method and GAPDH served as the internal reference. The primer sequences used in this study were shown below:

SIRT7

F: 5′-ACACCATTGTGCACTTTGG-3′

 R: 5′-CTTTAGAACCTTCAGGCTGG-3′

USP39

 F: 5′-ACCATTAACAGGAGTGTGCT-3′

 R: 5′-ACAGGCATAAGCATTGATGTG-3′

 FOXM1

 F: 5′-GACTTTGAAAGACATCTATACGTGG-3′

 R: 5′-GATGGAGTTCTTCCAGCCT-3′

 GAPDH

 F: 5′-TCATTTCCTGGTATGACAACGA-3′

 R: 5′-GGTCTTACTCCTTGGAGGC-3′.

### Subcellular fractionation

NE-PER™ Nuclear and Cytoplasmic Extraction Reagents (78,833, Thermo Fisher) were applied to isolate proteins in the cytoplasm and nucleus of transfected CSCC cells. In brief, cells were harvest with trypsin–EDTA and then centrifuged at 500 × g for 5 min. After washing cells with PBS, transferred cells to a 1.5 mL microcentrifuge tube and pellet by centrifugation at 500 × g for 2 min. Next, the cytoplasmic and nuclear proteins of the cells were extracted according to the kit instructions, and the isolated proteins were stored at − 80 °C for subsequent Western blot experiments to verify the expression of FOXM1.

### Western blot

RIPA lysis buffer (89900, Thermo Fisher) was used to isolate total proteins from tissue samples and cells. NE-PER™ Nuclear and Cytoplasmic Extraction Reagents (78833, Thermo Fisher) were applied to isolate proteins in the cytoplasm and nucleus of transfected CSCC cells. The protein samples were loaded onto 10% SurePAGE (M00667, GenScript) and electro-transferred onto PVDF membranes (1620177, Roche, Basel, Switzerland). The membranes were then blocked with 5% fat-free milk for 60 min at ambient temperature, and subsequently incubated overnight at 4 °C with the primary antibodies including anti-SIRT7 (ab259968, 1/1000, abcam), anti-LC3-I/II (ABC929, 1/500, Sigma-Aldrich), anti-USP39 (ab131244, 1/2000, abcam), anti-FOXM1 (ab207298, 1/1000, abcam), anti-H3 (ab1791, 1/1000, abcam) and β-actin (ab8226, 1/3000, abcam) as a loading control. Next day, Tris-buffered saline Tween-20 was used to wash the membranes three times and then subsequently incubated with corresponding secondary antibodies at ambient temperature for 60 min. Finally, the ECL chemiluminescent detection reagent was applied to visualize the proteins.

### Immunohistochemical (IHC) staining

Tumor tissues and adjacent normal tissues from cervical squamous cell carcinoma patients were fixated by 4% paraformaldehyde (FB002, Thermo Fisher) and dehydrated with gradient ethanol. Then the tissue samples were embedded in paraffin and sliced into 5 μm sections, followed by immunohistochemically staining with anti-SIRT7 (ab259968, 1/100, abcam) or anti-USP39 (ab131244, 1/100, abcam) [25]. A microscope (Olympus, Tokyo, Japan) was applied to capture the staining images and then the intensity of staining was evaluated to quantify the expression of SIRT7 and USP39.

### Colony formation assay

The transfected CaSki and SiHa cells were seeded into 6-well plates (1 × 10^4^ cells/well) and incubated in culture medium for 14 days. Next, the cell colonies were fixed using methanol (34860-1L-R, Sigma-Aldrich) for 30 min, followed by staining with 3% crystal violet solution (C0121, Beyotime) for 20 min. Finally, a microscope (Olympus, Tokyo, Japan) was applied for colony number calculation in five randomly chosen visual fields.

### Cell viability

CaSki and SiHa cell viability after indicated treatment was evaluated using a Cell Counting Kit-8 kit (C0037, Beyotime). The transfected CaSki and SiHa cells were plated into 96-well plates at 1500 cells/well. After culturing for 1, 2, 3 days, followed by supplementation with CCK-8 solution (10 μl), cells were further cultured for 60 min. A microplate reader (TECAN Spark 10 M, Shengyang, China) was applied to detect the OD values of cells at 450 nm.

### Cell apoptosis

Transfected CaSki and SiHa cells apoptosis was evaluated using an Annexin V-FITC/PI Apoptosis Kit (Mutisiences, China, AP101–100-kit). After PBS washing and centrifugation at 1000 rpm for 5 min, cells (1 × 10^6^) were re-suspended in 1X binding buffer. Cells were then stained with Annexin V/FITC solution (5 µL of FITC Annexin V) and 5 µL of propidium iodide (PI) mixture and incubated at RT for 30 min in the dark. CSCC cell apoptosis was evaluated by Gallios flow cytometry (BECKMAN COULTER).

### Transmission electron microscopy (TEM)

The formation of autolysosome in transfected CaSki and SiHa cells was assessed using TEM. CSCC cells were subjected to dihydrotestosterone (DHT) treatment for 72 h after steroid starvation for 2 days. Next, cells were gently scraped, followed by centrifugation and fixation with 2.5% glutaraldehyde in 0.1 M cacodylate buffer (pH 7.4) for 60 min at ambient temperature. Cells were then embedded and sliced to sections. (60 nm). Uranyl acetate and lead citrate were applied for section staining, and a JEM-1230 transmission electron microscopy (JEOL, Tokyo, Japan) was used for result analysis.

### mRFP-GFP-LC3 adenovirus infection assays

The autophagy of transfected CaSki and SiHa cells was measured using mRFP-GFP-LC3 adenovirus infection assays. Cells were transfected with GFP-LC3 adenoviruses (Hanbio, Shanghai, China) at a 20 multiplicity of infection (MOI) and incubated for 24 h. Then cells were cultured with Mito tracker Red for 20 min for autophagy measurement. A fluorescence microscope (Olympus) was applied to detect the fluorescent signals of cells in each transfection group.

### Mitochondrial membrane potential (MMP) measurement

The MMP of CaSki and SiHa cells in each group was determined by a Mitochondrial membrane potential assay kit with JC-1 (C2006, Beyotime). Transfected CaSki and SiHa cells (1 × 10^5^) were seeded in 6-well plates, and JC-1 dye was added and cultured for 20 min at 37 °C. Red fluorescence represented high MMP and green fluorescence indicated low MMP, which were detected using flow cytometry (examination wavelength: 488 nm, emission wavelength: 530 nm) [26].

### ROS production measurement

The ROS level of the transfected CSCC cells was determined by Reactive Oxygen Species Assay Kit (S0033S, Beyotime). The transfected CSCC cells were seeded into six-well plates at 1 × 10^5^ cells/well. After incubating for 24 h, DFCH-DA was used to treat the cells for 30 min at 37 °C in dark. The intracellular ROS levels were subjected to flow cytometry measurement and FlowJo software was applied for analysis of the results.

### Immunofluorescence

CaSki and SiHa cells were rinsed with PBS three times and fixated with 4% formalin at 25 °C for 15 min. After permeabilizing with 0.25% Triton X-100 (HFH10, Thermo Fisher) and blocking with blocking buffer for 30 min, CSCC cells were cultured with anti-SIRT7 (sc-365344, 1/100, SantaCruz) and anti-USP39 antibody (ab131244, 1/100, abcam) or anti-FOXM1 antibody (ab207298, 1/100, abcam) overnight at 4 °C. After rinsing with PBST (Thermo Fisher), CSCC cells were cultured with the corresponding fluorescent secondary antibodies for 60 min. DAPI (D9542-1MG, Sigma-Aldrich) was applied to counterstain the nucleus of CSCC cells. Finally, the subcellular location of SIRT7 and USP39 was observed with a fluorescence microscope (Olympus).

### Co-immunoprecipitation (Co-IP)

RIPA buffer was used to lyse the transfected CaSki and SiHa cells. The lysed cells were then subjected to centrifugation for 15 min at 12,000 × g. Immunoprecipitation was performed with Flag-USP39 and Myc-SIRT7. After culturing with protein-A agarose (sc-2001, Santa Cruz), the proteins were separated by 10% SurePAGE. The binding between USP39 and SIRT7 was evaluated by western blot using anti-Flag tag (ab205606, 1/3000, abcam) or anti-Myc (ab32, 1/3000, abcam) antibodies.

### In vitro* deacetylation assay*

CaSki cells were transfected with Flag-USP39, treated with 10 mM sirtuin inhibitor nicotinamide (NAM) (S1899, Selleck) or histone deacetylase (HDAC) inhibitor TSA (S1045, Selleck) for 6 h. After lysing cells in RIPA buffer, Flag-USP39 was bound to anti-Flag beads and incubated with Myc-SIRT7 for 60 min at 37 °C. USP39 acetylation levels were examined with anti-lysine acetylation antibodies. The acetylation level of USP39 was subjected to western blot analysis using anti-lysine acetylation antibodies.

### RNA immunoprecipitation (RIP)

The binding relation of USP39 protein and FOXM1 mRNA was examined using RIP assays. CaSki and SiHa cells were treated with RIP lysis buffer (Thermo Fisher) and subsequently cultured with sepharose beads conjugated with antibodies against USP39 (ab131244, abcam) or IgG (ab172730, abcam) overnight at 4 °C. The immunoprecipitated RNAs were extracted by culturing the samples with Proteinase K. The purified RNAs were subjected to qRT-PCR analysis.

### Chromatin immunoprecipitation (ChIP)

The interaction between FOXM1 and SIRT7 promoter was examined using ChIP assay. CaSki and SiHa cells (5 × 10^6^) were fixed with 1% formaldehyde (Sigma-Aldrich) for 10 min followed by ultrasonic treatment. The cell lysis was centrifuged at 13,000 rpm at 4 °C, and the supernatant was collected and cultured overnight at 4 °C with anti-FOXM1 (ab207298, abcam) or anti-IgG (ab172730, abcam). The endogenous DNA–protein complex was precipitated by Protein Agarose/Sepharose. After eluting by elution buffer (Beyotime), the enrichment of SIRT7 promoter fragment binding to FOXM1 was detected using qRT-PCR analysis.

### Dual-luciferase reporter assay

SIRT7 promoter region was subcloned into the pGL3-basic luciferase reporter vector (Promega, Madison, USA). CaSki and SiHa cells were grown in 24-well plate at 6 × 10^4^ cells/well and cultured for 24 h. Then cells were co-transfected with pGL3-SIRT7 promoter and empty pGL3 vectors with shNC, shUSP39#1 or shUSP39#1 + oe-FOXM1. 48 h later, dual Luciferase Reporter Assay System (Promega) was used to measure the relative luciferase reporter activities in each group normalized to Renilla luciferase activity following manufacturer’s protocol.

### Xenograft mouse models

Female BALB/c nude mice (4–5 weeks, 18 ± 5 g) were provided by the Vital River (Beijing, China). The animals were fed in SPF environment, with 12 h light/dark cycles at 25 °C and 60% humidity. Mice were randomly divided into the shNC and shSIRT7#1 groups (n = 6 per group). Xenograft mouse models were established by subcutaneously injecting transfected SiHa cells (shNC or shSIRT7#1, 10^7^ cells resuspended in 100 μL PBS) into left armpit of mice. The volume of mouse tumor was measured at day 7, 14, 21, 28 after modeling. The animals were anesthetized and then sacrificed by cervical dislocation on day 28 and mouse tumor weight was measured. The animal experimental protocol was approval by the Ethics Committee of our institution.

### Statistical analysis

GraphPad 8.0 was applied for data analysis and data were shown as the mean ± standard deviation. The difference between the two groups was analyzed using Student’s t-test, and the one-way analysis of variance (ANOVA) was applied for difference assessment among multiple groups. A P-value less than 0.05 indicated statistical significance.

## Results

### SIRT7 is highly expressed in CSCC patient tissues and promotes CSCC tumorigenesis

**Fig. 1:**
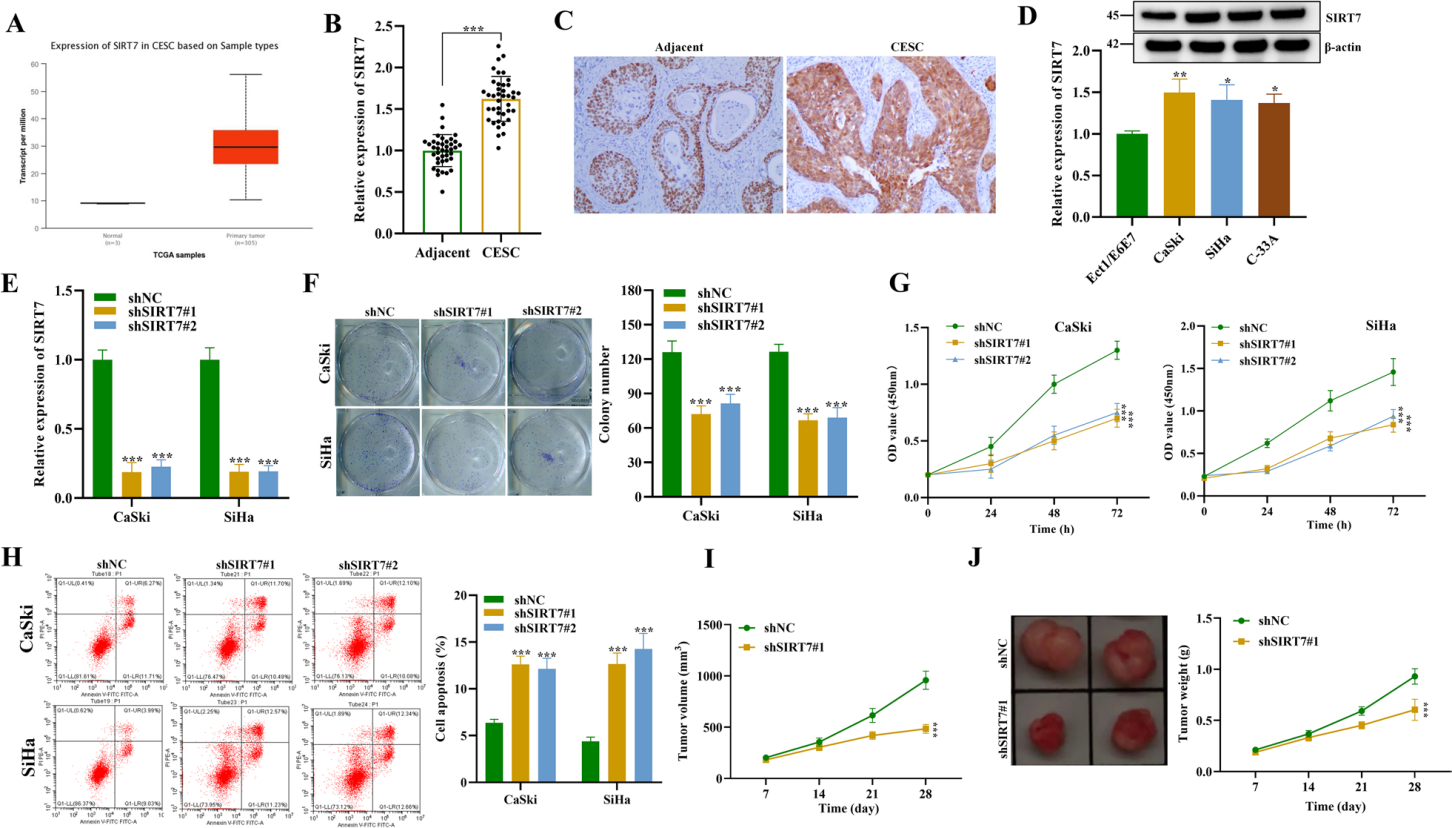
SIRT7 promotes cervical squamous cell carcinoma tumorigenesis both in vitro and in vivo. **A** The expression pattern of SIRT7 in cervical squamous cell carcinoma (CSCC) (n = 305) and normal tissue (n = 3) was predicted using the UALCAN database (http://ualcan.path.uab.edu/). **B** SIRT7 mRNA levels in the tumor tissues (n = 40) of CSCC patients and normal adjacent tissues (n = 40) were measured using qRT-PCR analysis. **C** IHC analysis was performed to determine the protein levels of SIRT7 in CSCC tissues and normal tissues. **D** qRT-PCR and western blot analysis were used to detect the mRNA and protein expression levels of SIRT7 in human cervical squamous cell carcinoma cell lines (CaSki, SiHa, C-33A) and human normal cervical epithelial cells (Ect1/E6E7 cells). **E** SIRT7 expression in CaSki and SiHa cells after the transfection of shSIRT7#1/#2 was measured using qRT-PCR. **F** Colony formation assay was conducted to evaluate the proliferation of CSCC cells after silencing SIRT7. **G** CCK-8 assay was used to detect the viability of CSCC cells transfected with shSIRT7#1/#2. **H** Flow cytometry was used to assess the apoptosis rate of CSCC cells after SIRT7 knockdown. **I** Tumor volume of mice injected with transfected SiHa cells (shNC or shSIRT7#1) (n = 6 per group). **J** The images of mouse tumors and the tumor weight in the shNC and shSIRT7 groups. All results are representative of at least 3-independent experiments. *p < 0.05, **p < 0.01, ***p < 0.001

We first evaluated the expression level of SIRT7 in cervical squamous cell carcinoma (CSCC) tumor and normal tissues using the UALCAN database (http://ualcan.path.uab.edu/index.html). As revealed by the UALCAN analysis, SIRT7 mRNA levels were significantly upregulated in the tumor tissues of CSCC patients relative to normal tissues (Fig. 1A). SIRT7 expression in the tumor tissues of CSCC patients (n = 40) and normal adjacent tissues (n = 40) was also measured using qRT-PCR analysis. The upregulation of SIRT7 in CSCC tissue was also identified in patients’ tissues compared to the normal tissue samples (Fig. 1B). Furthermore, the relationship between SIRT7 expression and tumor characteristics in patients with CSCC was analyzed through the clinical data of 305 CSCC patients in the TCGA database. Chi-square test results revealed that the expression of SIRT7 was positively correlated with the grade of cervical squamous cell carcinoma and nodal metastasis status (Table 1). These results were further confirmed by IHC staining. According to the results of IHC analysis, SIRT7 protein was also expressed at high levels in CSCC tissue samples (Fig. 1C). We then verified these results using different cell lines. qRT-PCR and western blot analyses also validated the upregulation of SIRT7 protein levels in cervical squamous cell carcinoma cells (CaSki, SiHa, C-33A) compared with the normal Ect1/E6E7 cells (Fig. 1D). To investigate the functional role of SIRT7 in CSCC tumorigenesis, we silenced SIRT7 expression in CaSki and SiHa cells by transfecting SIRT7 shRNA. Results demonstrated that silencing of SIRT7 dramatically decreased its expression level in CSCC cells (Fig. 1E). The effects of SIRT7 deficiency on cell malignancy in CSCC was investigated. We found that the colony number of CSCC cells was significantly reduced after silencing SIRT7 (Fig. 1F). Similarly, the viability of CSCC cells was significantly reduced in the shSIRT7#1/#2 groups compared with the shNC group (Fig. 1G). Moreover, SIRT7 knockdown also showed significant enhancement on the apoptosis of CSCC cells (Fig. 1H). To further validate the tumorigenic role of SIRT7 in vivo, Xenograft mouse models were established. As shown in Fig. 1I, we observed decreased tumor volume after SIRT7 silencing, and the growth rate of mouse tumor was lower after SIRT7 knockdown. Moreover, the tumor images revealed that SIRT7 silencing decreased mouse tumor size and weight compared with the shNC group (Fig. 1J). In addition, we measured apoptosis levels using flow cytometry in mouse tumors. The results showed that apoptosis increased significantly after SIRT7 knockdown (Additional file 1: Fig S1A). Western blot showed that the expression levels of FOXM1 and USP39 diminished with the knockdown of SIRT7 expression, and the expression of autophagy protein LC3-II/LC3-I also decreased significantly after SIRT7 knockdown (Additional file 1: Fig S1B). Moreover, the ROS levels in mouse tumors underwent significant increase after SIRT7 knockdown as evident from DCFH-DA staining (Additional file 1: Fig S1C). Taken together, these results suggest that SIRT7 is highly expressed in CSCC and promotes its tumorigenesis.

**Table 1:** Relationship between SIRT7 expression and tumor characteristics in patients with high-grade glioma

Features	No. of patients	SIRT7 expression		χ^2^, p-value
		High	Low	
All patients	305	215	90	
Individual cancer stages				20.59, 0.0001
Stage1	166	100	66	
Stage2	69	54	15	
Stage3	46	38	8	
Stage4	23	22	1	
Tumor grade				3.037, 0.386
Grade1	43	28	15	
Grade2	135	102	33	
Grade3	118	79	39	
Grade4	9	6	3	
Nodal Metastasis status				8.127, 0.0044
N0	179	115	64	
N1	126	100	26	

### SIRT7 knockdown inhibits autophagy to promote ROS accumulation

**Fig. 2:**
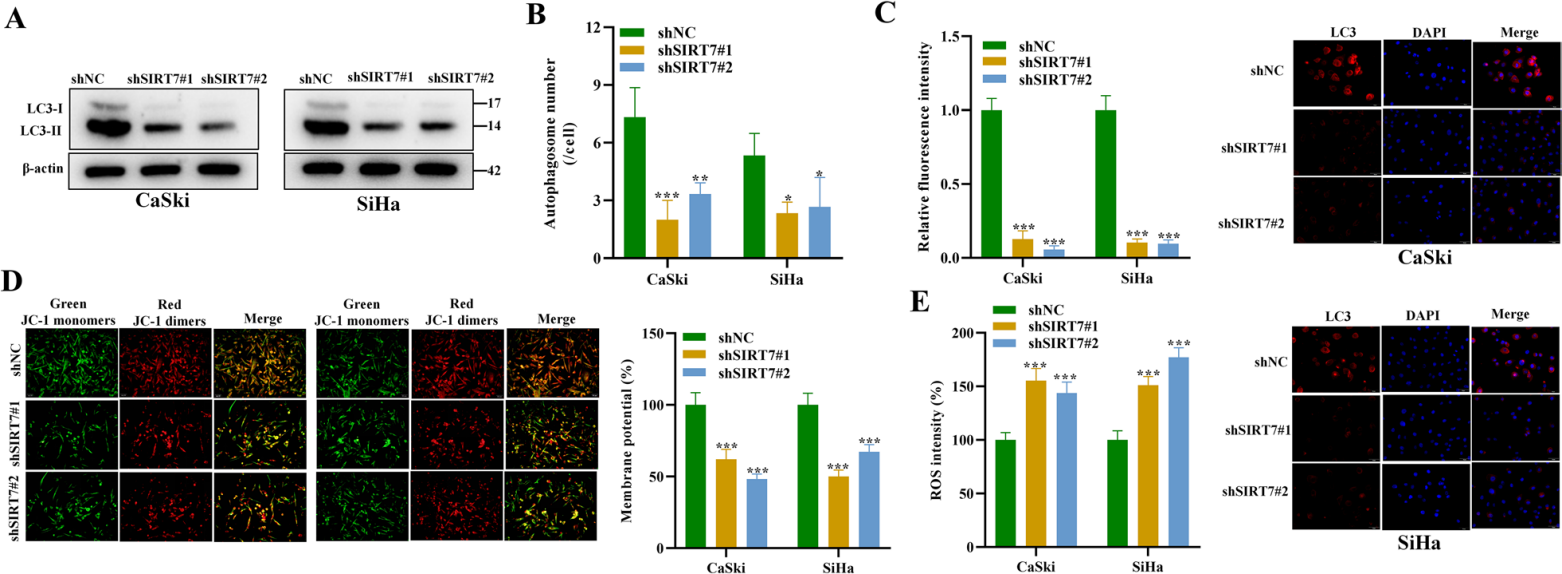
SIRT7 knockdown inhibits autophagy to promote ROS accumulation. **A** Western blot was used to detect the key proteins related to autophagy (LC3-I, LC3-II) in CaSki and SiHa cells after transfection of shSIRT7#1/#2. **B** TEM was used for the observation and quantification of autophagosomes in each group. **C** The mRFP-GFP-LC3 adenovirus infection assay was performed to examine the autophagy in CSCC cells. **D** JC-1 staining assay was used to detect the mitochondrial membrane potential of CSCC cells after indicated transfection. **E** DCFH-DA staining was used to detect the ROS levels in CSCC cells after indicated transfection. All results are representative of at least 3-independent experiments. *p < 0.05, **p < 0.01, ***p < 0.001

Previously, it has been shown that SIRT7 is involved in promoting cancer cell autophagy [12]. Therefore, we wanted to investigate the impact of SIRT7 on CSCC cell autophagy. The results of western blot analysis demonstrated that SIRT7 silencing significantly reduced the LC3-II/LC3-I levels (Fig. 2A). According to the observation using TEM, the number of autophagosomes showed a significant reduction after SIRT7 knockdown (Fig. 2B). In line with this, the results of mRFP-GFP-LC3 adenovirus infection assays revealed that SIRT7 silencing significantly reduced the formation of LC3 punctate, suggesting that SIRT7 facilitated the autophagic flux of CSCC cells (Fig. 2C). These findings showed that SIRT7 promoted autophagy of CaSki and SiHa cells. The autophagy of cancer cells protects cells from various stress, and the accumulation of ROS leads to mitochondrial damage when autophagy is inhibited. Thus, we next detected the impact of SIRT7 silencing on ROS levels in CSCC cells. We found that the membrane potential of mitochondria was significantly reduced after SIRT7 knockdown (Fig. 2D). Moreover, according to DCFH-DA staining, the ROS levels in CSCC cells underwent significant increase after SIRT7 knockdown (Fig. 2E). Overall, SIRT7 promotes the autophagy to inhibit the ROS accumulation of CSCC cells.

### SIRT7 interacts with and mediates USP39 deacetylation

**Fig. 3:**
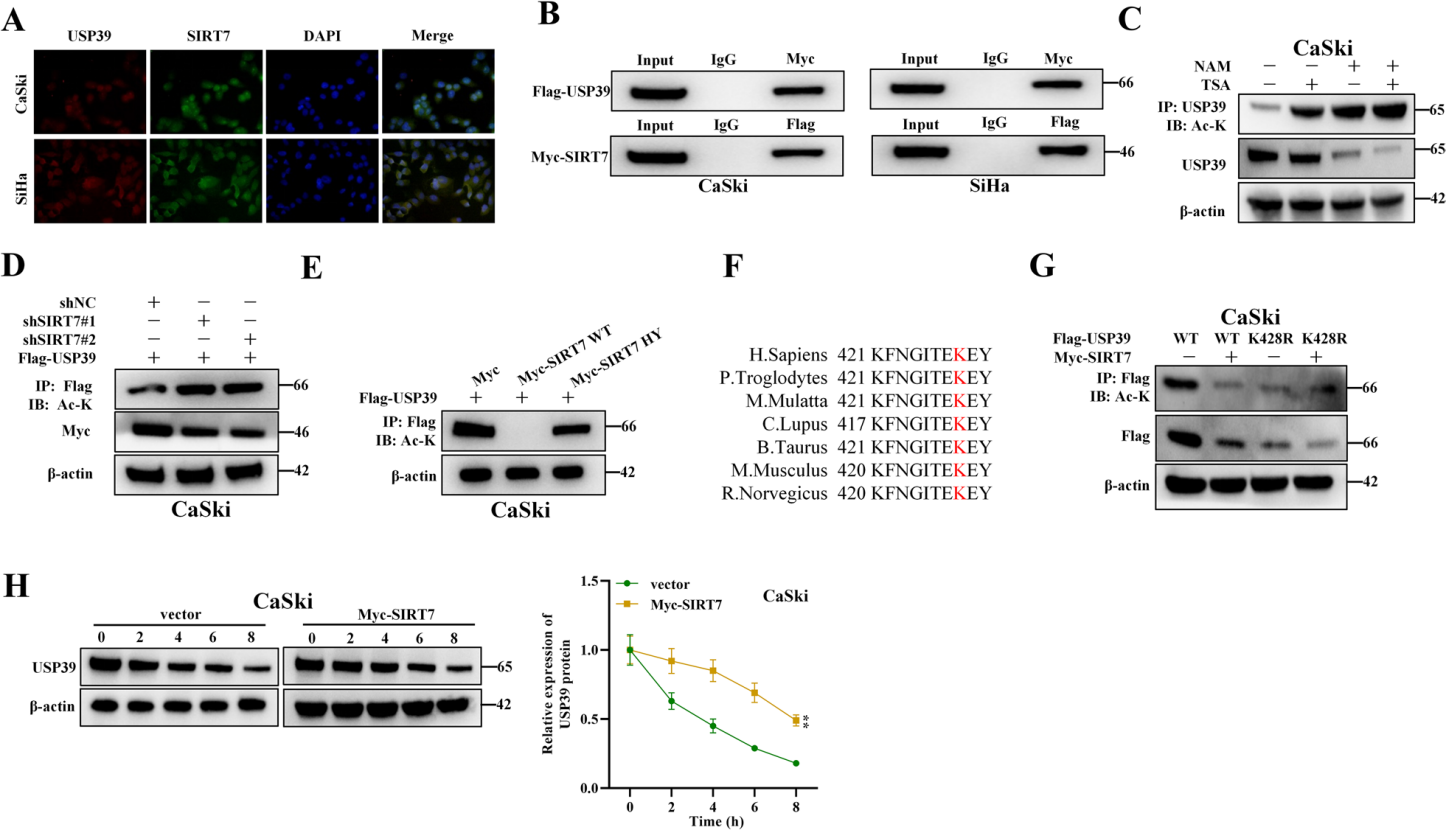
SIRT7 interacts with and mediates USP39 deacetylation. **A** IF staining was used to detect the subcellular location of SIRT7 and USP39 in CaSki and SiHa cells. **B** Co-IP assays were conducted to evaluate the interaction between USP39 and SIRT7 in CaSki and SiHa cells. **C** USP39 acetylation levels were examined by IP-WB in CaSki and SiHa cells treated with NAM or TSA inhibitors. **D** IP-WB was used to detect the acetylation level of USP39 in SIRT7 silenced CaSki cells. **E** The USP39 acetylation levels in CaSki cells with wild-type or mutant SIRT7 (SIRT7 H187Y) were detected by WB. **F** Sequence alignment of conserved K428-containing regions in USP39 orthologs of multiple species. **G** IP-WB assays were used to determine the acetylation level of USP39 in CaSki cells with wild-type or mutant USP39. **H** Western blot was performed to measure the protein expression of USP39 in CaSki cells after CHX treatment in indicated groups. All results are representative of at least 3-independent experiments. **p < 0.01

Next, we wanted to explore whether SIRT7 mediate its tumorigenic function via interacting with USP39. For this purpose, we first evaluated the co-localization of SIRT7 and USP39 in CSCC cells. According to IF staining, SIRT7 and USP39 both were colocalized in the nucleus of CaSki and SiHa cells (Fig. 3A). The results of Co-IP revealed that Flag-USP39 was significantly enriched in the precipitates of Myc-SIRT7 and vice versa, which indicated that USP39 interacted with SIRT7 in CaSki and SiHa cells (Fig. 3B). A previous study suggested that SIRT7 promoted the deacetylation of USP39 in hepatocellular carcinoma cells [24]. Therefore, we assessed if USP39 acetylation was affected by SIRT7 in cervical cancer cells. After treatment with histone deacetylase (HDAC) inhibitor TSA or SIRT inhibitor NAM, USP39 acetylation level exhibited a significant elevation. The combination of NAM and TSA had a stronger effect than NAM or TSA alone (Fig. 3C). Then acetylation level of USP39 was also examined under the transfection of sh-SIRT7 in CaSki cells, and the results demonstrated that USP39 acetylation levels were significantly elevated by sh-SIRT7 (Fig. 3D). Further analysis revealed that USP39 acetylation was inhibited by wild type SIRT7, and the mutant SIRT7 without USP39 acetylation activity (SIRT7 H187Y) was demonstrated to suppress USP39 deacetylation (Fig. 3E). Previous report indicates that SIRT7 interacts with the C-terminal ubiquitin hydrolase domain of USP39 [24]. Therefore, we next evaluated the binding site between SIRT7 and USP39. Based on the analysis of PhosphoSitePlus website, we found an acetylation site K428 in this domain. As shown in Fig. 3F, the K428 site was conservative in multiple species. The acetylation level of USP39 decreased significantly in the USP39 WT group after the presence of SIRT7, while the acetylation level of the USP39 mutant K428R group did not change significantly, indicating that SIRT7 mediates USP39 deacetylation at the site K428 (Fig. 3G). To further determine whether SIRT7 can regulate the stability of USP39 protein, we next examined USP39 protein levels in the presence of cycloheximide (CHX), an inhibitor of protein synthesis. After CHX treatment, the stability of USP39 protein levels were evidently lower in the Myc-SIRT7 groups (Fig. 3H). In addition, we also evaluated the interaction between SIRT7 and USP39 in human CSCC tissues. Co-immunoprecipitation indicated that SIRT7 interacted with USP39 (Additional file 1: Fig S2A). These results indicate that SIRT7 promotes the stability of USP39 via deacetylation.

### USP39 acts as tumor promoter in CSCC

**Fig. 4:**
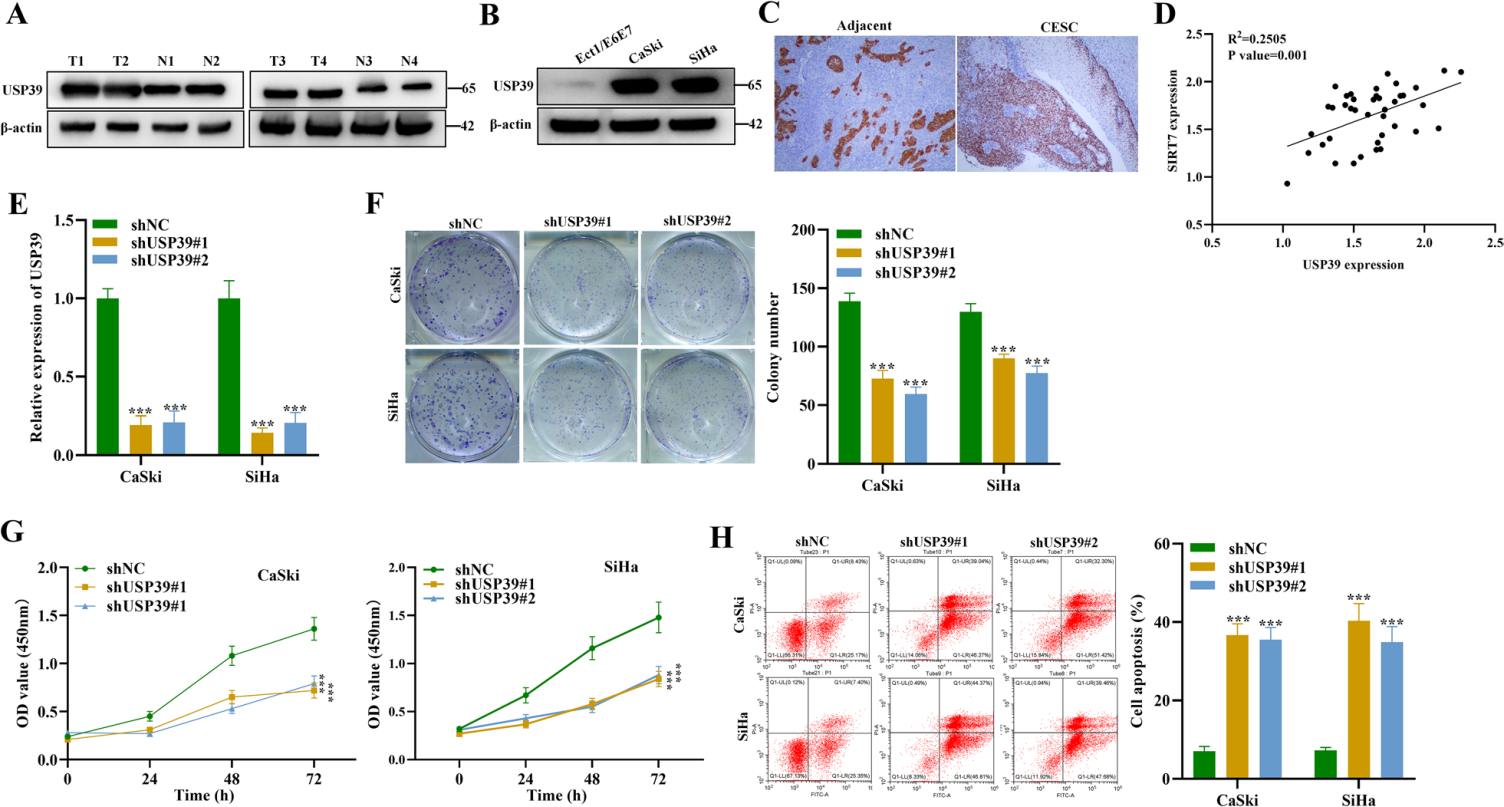
USP39 promotes tumorigenesis in CSCC. **A** Western blot was used to detect the protein expression of USP39 in tumor tissues and adjacent normal tissues of cervical squamous cell carcinoma patients. **B** The protein level of USP39 in human cervical squamous cell carcinoma cell lines (CaSki, SiHa) and human normal cervical epithelial cells (Ect1/E6E7) was detected using western blot assay. **C** IHC assay was performed to detect the protein expression of USP39 in the normal and tumor tissues of cervical squamous cell carcinoma patients. **D** The correlation analysis was performed between the expression of SIRT7 and USP39 in the tumor tissues of cervical squamous cell carcinoma patients. **E** The knockdown efficiency of USP39 in CaSki and SiHa cells was assessed using qRT-PCR analysis. **F** Colony formation and (**G**) CCK-8 assays were conducted to evaluate the proliferation potential and viability of CaSki and SiHa cells transfected with shUSP39#1/#2. **H** The apoptosis rate of CaSki and SiHa cells after silencing USP39 was measured by flow cytometry. All results are representative of at least 3-independent experiments. ***p < 0.001

After confirming that SIRT7 and USP39 interacts with each other and that SIRT7 facilitates USP39 stability via deacetylation, we next evaluated the expression level and functional role of USP39 in CSCC tumorigenesis. According to western blot analysis, the protein expression of USP39 was highly expressed in CSCC tissue samples and cells compared to the normal tissues and cells (Fig. 4A, B). Similarly, IHC assay also identified the higher USP39 expression in the tumor tissues of CSCC patients (Fig. 4C). We the evaluated the correlation between SIRT7 and USP39 and a positive correlation between the expression of SIRT7 and USP39 was verified in the tumor tissues of CSCC patients (Fig. 4D). Then we wanted to explore the functional role of USP39 in CSCC tumorigenesis. We first silenced USP39 in CSCC cells, and USP39 mRNA expression exhibited significant reduction in CaSki and SiHa cells transfected with shUSP39#1/#2 (Fig. 4E). The impact of USP39 on CSCC cell proliferation potential and viability was investigated. Results demonstrated that USP39 deficiency showed significant inhibition on CSCC cell colony formation and viability (Fig. 4F, G). Moreover, CSCC cell apoptosis exhibited a significant increase after silencing USP39 (Fig. 4H). Overall, the results suggest that USP39 facilitates cell proliferation and decreases cell apoptosis rate in cervical squamous cell carcinoma.

### *USP39 regulates SIRT7 expression *via* alternative splicing of FOXM1 pre-mRNA*

**Fig. 5:**
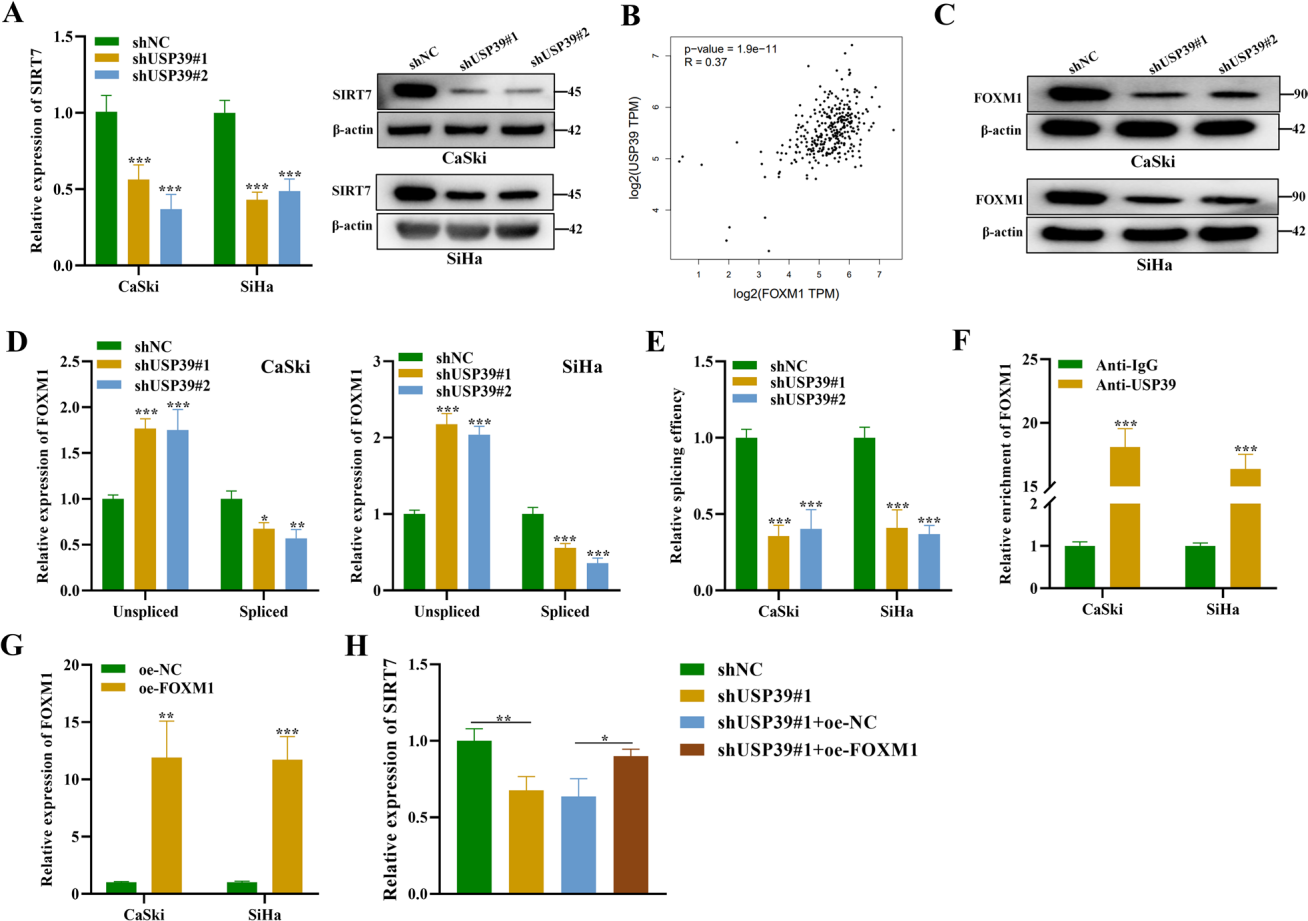
USP39 regulates alternative splicing of FOXM1 pre-mRNA. **A** qRT-PCR and western blot analyses were performed to detect the mRNA and protein levels of SIRT7 in CaSki and SiHa cells transfected with shUSP39#1/#2. **B** The correlation of USP39 and FOXM1 in the cervical squamous cell carcinoma tissues was predicted using the GEPIA database (http://gepia.cancer-pku.cn/). GEPIA database sources CESC tumor and CESC normal samples in the TCGA database. **C** Western blot was used to detect FOXM1 protein levels in CaSki and SiHa cells after USP39 knockdown. **D** qRT-PCR analysis was used to measure the mRNA expression of spliced and unspliced FOXM1 RNA transcripts and (**E**) Splicing efficacy in CaSki and SiHa cells transfected with shUSP39#1/#2. **F** The interaction between FOXM1 and USP39 was explored using RIP assay. **G** qRT-PCR was used to evaluate the overexpression efficacy of FOXM1 in CaSki and SiHa cells. **H** The expression of SIRT7 in cervical squamous cell carcinoma cells after indicated transfections were measured using qRT-PCR analysis. All results are representative of at least 3-independent experiments. *p < 0.05, **p < 0.01, ***p < 0.001

In the above experiment, we have shown that SIRT7 interacts with USP39 and enhance protein stability of USP39 via deacetylation and that USP39 regulates SIRT7 expression. Next, we investigated the potential impact of USP39 silencing on the expression of SIRT7 mRNA and protein was explored. Indeed, we noticed that SIRT7 mRNA and protein expression levels were significantly reduced in USP39 silenced CSCC cells (Fig. 5A). Based on this, we next wanted to explore the molecular mechanism by which SIRT7 and USP39 regulates and mediate its function in CSCC. A previous study has indicated that USP39 has no significant impact on SIRT7 ubiquitination. Hence, it was assumed that USP39 might regulate SIRT7 via other mechanisms. USP39 has been reported to positively regulate FOXM1 expression in hepatocellular carcinoma cells [27]. Interestingly, the expression of FOXM1 and SIRT7 is demonstrated to be correlated in gastric cancer, and FOXM1 deficiency downregulates SIRT7 in gastric cancer cells [28]. To this end, we first explored the correlation between USP39 and FOXM1. According to the GEPIA analysis, the expression of USP39 and FOXM1 was in positive correlation in the cervical squamous cell carcinoma (R = 0.36, p-value = 1e-10) (Fig. 5B). This data was further confirmed by western blot analysis that showed a significant downregulation of FOXM1 induced by USP39 knockdown in CSCC cells (Fig. 5C). Moreover, unspliced and spliced expression of FOXM1 transcript was assessed using qRT-PCR. It was observed that unspliced FOXM1 transcript expression was significantly upregulated by USP39 knockdown, while the expression of spliced FOXM1 exhibited a significant decrease after silencing USP39 (Fig. 5D). After that, we measured the splicing efficacy of FOXM1 transcript in USP39 silenced CaSki and SiHa cells, and the results indicated that USP39 silencing showed significant inhibition on the splicing of the pre-mRNA of FOXM1 (Fig. 5E). Results of RIP assay further verified this data and revealed the abundant enrichment of FOXM1 in the precipitates of anti-USP39, suggesting that FOXM1 bound with USP39 in CaSki and SiHa cells (Fig. 5F). Then FOXM1 overexpression efficacy was verified using qRT-PCR analysis (Fig. 5G). Moreover, SIRT7 expression was revealed to be downregulated after USP39 silencing, which was reversed after FOXM1 overexpression in cervical squamous cell carcinoma cells (Fig. 5H). We also used RIP assay to confirm that FOXM1 bound with USP39 in human CSCC tissue (Additional file 1: Fig S2B). Overall, these finding suggest that USP39 regulates SIRT7 expression by modulating FOXM1.

### USP39 promotes SIRT7 expression by activating the transcriptional activity of FOXM1

**Fig. 6:**
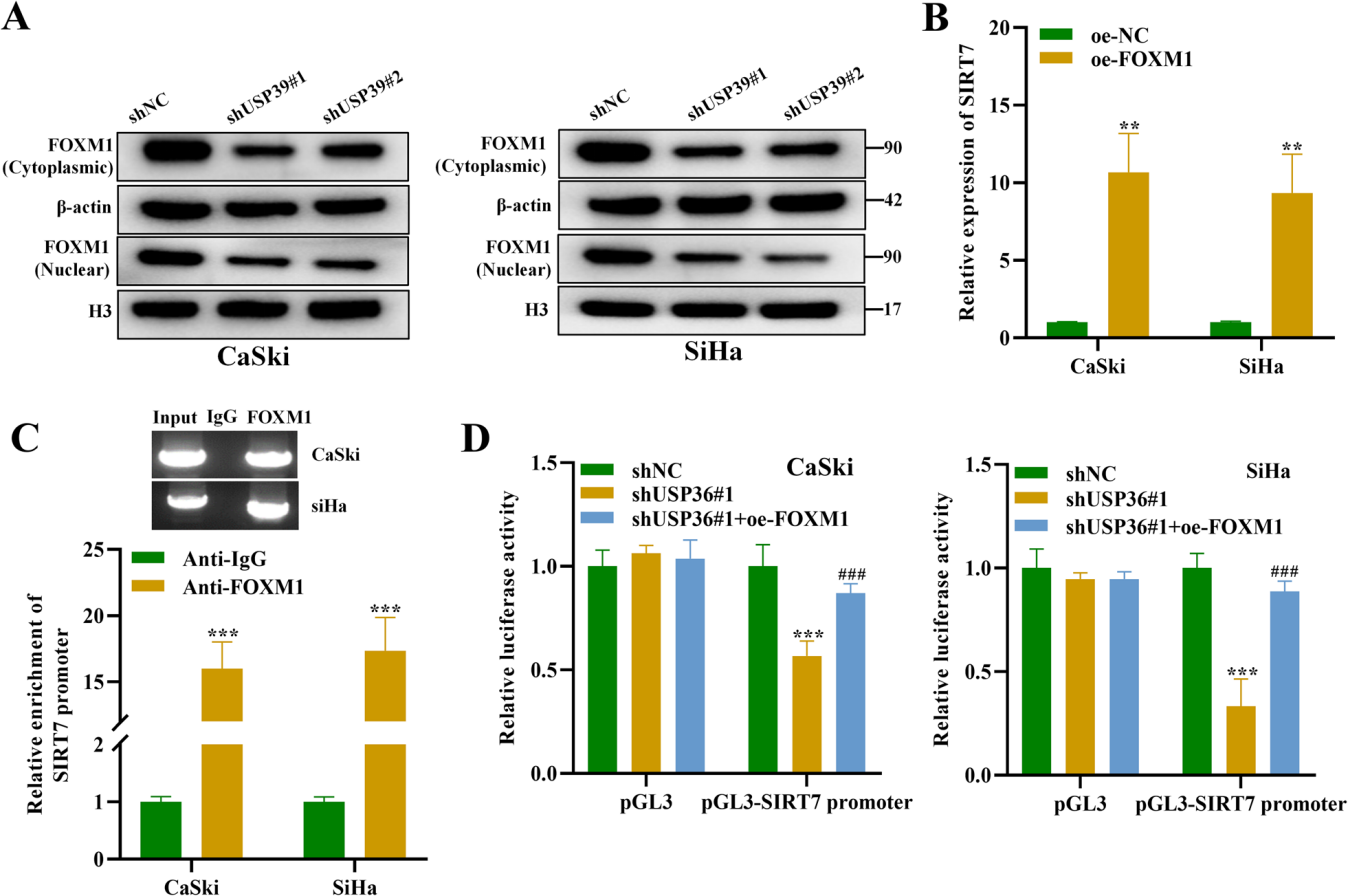
USP39 promotes SIRT7 expression by activating the transcriptional activity of FOXM1. **A** Western blot was used to detect the protein expression of FOXM1 in the cytoplasm and nucleus of CaSki and SiHa cells transfected with shUSP39#1/#2. **B** qRT-PCR was used to measure the mRNA expression of SIRT7 after overexpressing FOXM1 in CaSki and SiHa cells. **C** ChIP assay was performed to explore the interaction between FOXM1 and SIRT7 in CaSki and SiHa cells. **D** Dual-luciferase reporter assays were conducted to measure the luciferase activity of the pGL3-SIRT7 promoter in CaSki and SiHa cells after indicated transfection. All results are representative of at least 3-independent experiments. **p < 0.01, ***p < 0.001; ###*p* < 0.001

We further explored the role of FOXM1 in regulating the expressions of USP39 and SIRT7. The cytoplasmic and nuclear FOXM1 protein levels in CSCC cells were explored. We separated cell cytoplasm and nuclei by NE-PER™ Nuclear and Cytoplasmic Extraction Reagents. Then we used Western blot to detect the expression levels of FOXM1 in nuclei and cytoplasmic proteins respectively, and the proteins of the cytoplasm and nuclei were quantified using β-actin and H3. We found that USP39 silencing led to a decrease in FOXM1 expression at protein levels in CSCC cell nuclei (Fig. 6A). Then we measured the impact of FOXM1 on SIRT7 level in CSCC cells, and we found a dramatic elevation in SIRT7 expression after overexpressing FOXM1 (Fig. 6B). Furthermore, ChIP assay showed that SIRT7 promoter was abundantly enriched in the precipitates of anti-FOXM1, suggesting that FOXM1 bound with SIRT7 promoter (Fig. 6C). According to dual-luciferase reporter assays, luciferase reporter activities of pGL3-SIRT7 promoter were significantly reduced after USP39 silencing, which was reversed after FOXM1 overexpression in CaSki and SiHa cells (Fig. 6D). In addition, ChIP assay showed that SIRT7 promoter was rich in anti-FOXM1 precipitate, indicating that FOXM1 was bound to the SIRT7 promoter in human CSCC tissue (Additional file 1: Fig S2C). Taken together, these results indicate that USP39 promotes SIRT7 transcription by elevating the transcriptional activity of FOXM1.

### *SIRT7 regulates oxidative stress in cervical squamous cell carcinoma *via* USP39 and FOXM1*

**Fig. 7:**
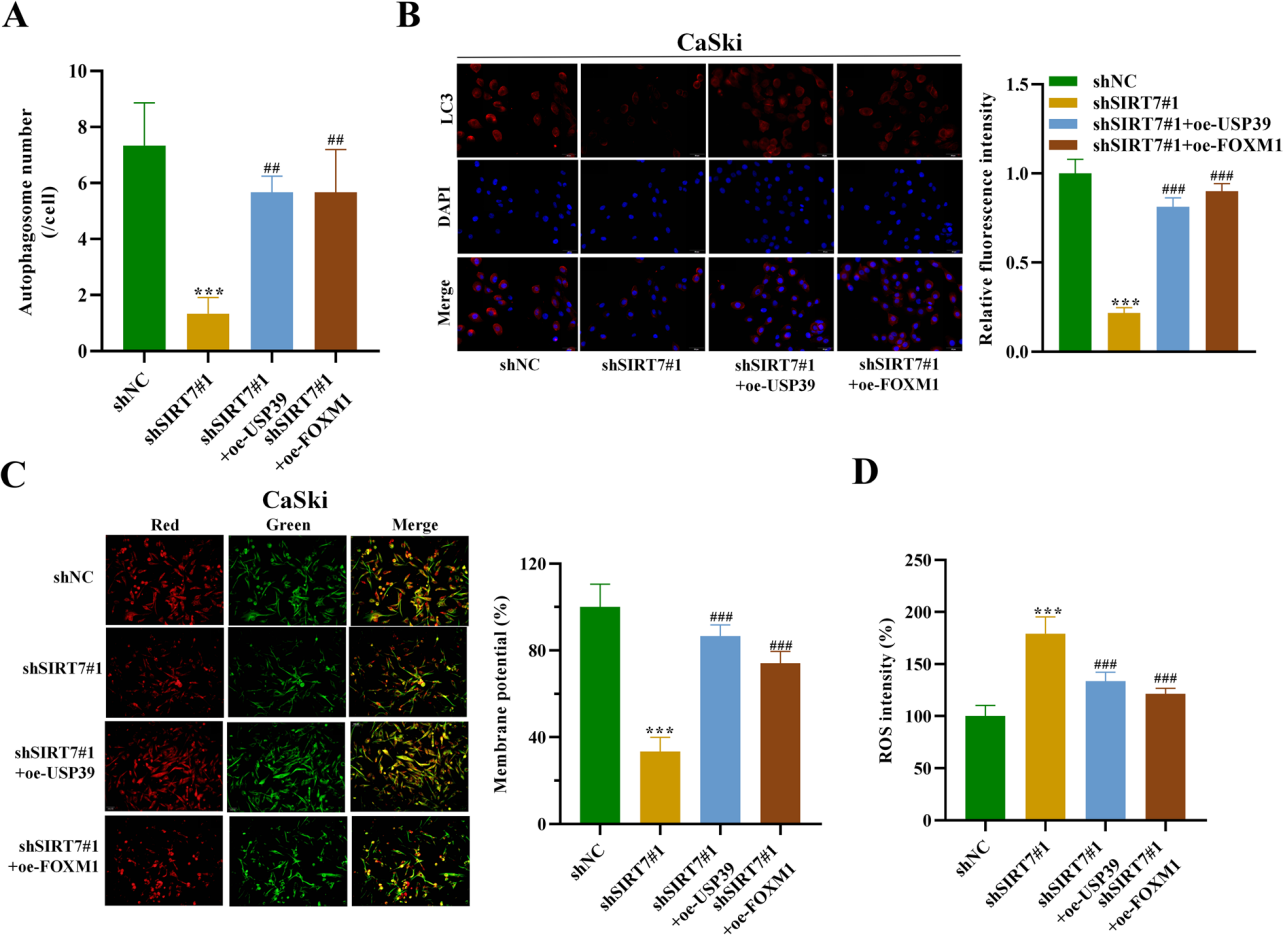
SIRT7 regulates oxidative stress in cervical squamous cell carcinoma via USP39 and FOXM1. **A** TEM was applied to observe and calculate the number of autophagosomes in CaSki cells in each group. **B** The mRFP-GFP-LC3 adenovirus infection assay was used to examine the autophagy levels of CaSki cells in each group. **C** JC-1 staining assays were used to detect the mitochondrial membrane potential (MMP) of CaSki cells after indicated transfection. When the MMP was high, JC-1 produced red fluorescence. When the MMP was low, JC-1 produced green fluorescence. **D** DCFH-DA staining assays were used to determine the ROS levels in CaSki cells in each group. All results are representative of at least 3-independent experiments. ***p < 0.001; ##*p* < 0.01, ###*p* < 0.001

Finally, we wanted to explore whether SIRT7 regulates oxidative stress in cervical squamous cell carcinoma via USP39 and FOXM1. For this purpose, the CaSki cell autophagosomes were analyzed under TEM. Interestingly, their numbers showed a significant decrease after SIRT7 silencing, which was revealed to be reversed after USP39 or FOXM1 overexpression (Fig. 7A). Based on the results of mRFP-GFP-LC3 adenovirus infection assays, we also found that the reduction in the formation of LC3 punctate induced by SIRT7 silencing was reversed after USP39 or FOXM1 overexpression, suggesting that SIRT7 facilitated the autophagy of cervical squamous cell carcinoma cells via USP39 and FOXM1 (Fig. 7B). Moreover, the results of JC-1 staining assay further demonstrated that the mitochondrial membrane potential was significantly decreased after silencing SIRT7, which was revealed to be counteracted by USP39 or FOXM1 overexpression in CaSki cells (Fig. 7C). Additionally, the results of DCFH-DA staining assay also showed that SIRT7 silencing induced an increase in ROS levels in CaSki cells which was reduced by USP39 or FOXM1 overexpression (Fig. 7D). Collectively, these findings indicate that SIRT7 promotes autophagy and inhibits ROS accumulation in CSCC cells via USP39 and FOXM1.

## Discussion

In the present study, we elucidated the function and regulatory mechanism of SIRT7 in CSCC progression. SIRT7 was highly expressed in CSCC tissue samples and cells, and SIRT7 silencing significantly repressed CSCC cell growth and autophagy in vitro and tumorigenesis in vivo. USP39 was deacetylated by SIRT7 and promoted SIRT7 expression by facilitating FOXM1-mediated SIRT7 transcription. Furthermore, the SIRT7/USP39/FOXM1 positive feedback loop was demonstrated to promote the autophagy and suppress the oxidative stress in CSCC.

SIRT7 is a NAD^+^-dependent deacetylase that regulates gene expression by deacetylating histones [7]. Accumulating evidence has revealed that SIRT7 exerts oncogenic effect in various malignancies including prostate cancer, hepatocellular carcinoma, and cholangiocarcinoma [12, 29, 30]. SIRT7 is also shown to regulate the cell proliferation and survival in cancer progression. Autophagy is a cellular process linked with various pathologies such as infection, aging and cancer [31]. Cancer cell autophagy is suggested to maintain mitochondrial function, and accumulation of ROS may lead to mitochondrial damage when autophagy is inhibited [32, 33]. Oxidative stress is a primary pathophysiological mechanism in a variety of human diseases. The excessive ROS can induce mitochondrial mediated apoptosis [34, 35]. Sirtuins are reported to regulate autophagy and oxidative stress in cancer [36]. SIRT7 is revealed to promote the autophagy in cancer progression [12]. Accordingly, in our work, we also observed that SIRT7 deficiency repressed the autophagy of CSCC. MMP levels were significantly decreased while the ROS levels were evidently elevated in CSCC cells after SIRT7 knockdown. Also, we noticed that SIRT7 promoted autophagy while inhibited ROS accumulation in cervical squamous cell carcinoma cells.

SIRT7 promotes hepatocellular carcinoma development via deacetylation of USP39 [24]. USP39 is revealed to be implicated in assembly of the RNA spliceosome and play a critical role in mRNA splicing [19, 20]. Moreover, USP39 upregulation is identified in the pathogenesis of multiple cancers, including hepatocellular carcinoma, medullary thyroid carcinoma and renal cell carcinoma [27, 37, 38]. In this study, we found that SIRT7 and USP39 were colocalized in the CSCC cell nucleus. SIRT7 inhibition significantly elevated the acetylation level of USP39. SIRT7 was demonstrated to deacetylate USP39 to enhance its protein stability at K428 site. Moreover, the impact of USP39 on CSCC malignant cell behaviors was explored. USP39 protein level showed significant elevation in CSCC cells and tissue samples. SIRT7 and USP39 expression was found positively correlated in the tissue samples of CSCC. Additionally, USP39 silencing was demonstrated to suppress cell proliferation potential and promote cell apoptosis in cervical squamous cell carcinoma in vitro.

USP39 has been reported to positively regulate FOXM1 expression in hepatocellular carcinoma [27]. FOXM1 belongs to the Forkhead family of transcription factors and is previously demonstrated to be an oncogene in various malignancies [39–41]. It has also been revealed to be correlated to the expression of SIRT7 in gastric cancer [28]. In our work, USP39 interacted with FOXM1 and promoted the splicing of FOXM1 pre-mRNA. Furthermore, the reduction in SIRT7 expression induced by USP39 silencing was reversed after FOXM1 overexpression, which suggest that USP39 promoted the expression of SIRT7 through FOXM1. USP39 silencing also caused significant decrease in FOXM1 protein expression in CSCC cells. FOXM1 overexpression was demonstrated to upregulate SIRT7. SIRT7 promoter region was found to be bind with FOXM1 in cervical squamous cell carcinoma cells. These results suggested that USP39 promoted the transcriptional activity of FOXM1 to promote SIRT7 transcription. This positive feedback was then further verified using rescue assays, which indicated that indeed SIRT7 promoted the autophagy and inhibited ROS production through USP39 and FOXM1 in cervical squamous cell carcinoma. These results are in agreement with the findings of above-mentioned studies.

In conclusion, our key findings from this report demonstrate that SIRT7 facilitates the proliferation, autophagy and tumor growth while inhibits the apoptosis and ROS production in CSCC. By using mouse model approach, we also provide evidence that SIRT7 knockdown inhibited the tumor growth. SIRT7 promotes USP39 protein stability via deacetylation at the site K428, and USP39 facilitates the splicing efficacy of FOXM1 to elevate FOXM1 expression. Furthermore, FOXM1 function as a transcriptional factor that positively regulates SIRT7 expression. This SIRT7/USP39/FOXM1 positive feedback loop promotes the autophagy and suppresses the oxidative stress in CSCC (Graphical Abstract). SIRT7/USP39/FOXM1 is highly expressed in CSCC, but whether the pathogenesis in cervical squamous cell carcinoma patients is inhibited by acetylation of USP39 at the k428 site still needs further study. If accurate, a unique inhibitor might be created as a therapy option for CSCC. This newly identified SIRT7/USP39/FOXM1 axis might serve as a novel prognostic biomarker and an effective targeted therapy for CSCC treatment. In addition, CSCC cells (CaSki, SiHa) were mainly used for verification in this study, and the human CSCC tissues were used for follow-up experiments to a limited extent, and no mechanism analysis was done in human CSCC primary cells. In the future, the therapeutic influence of specific inhibitors on CSCC tumor tissue genesis will be explored by analyzing the effect of USP39-specific acetylation on SIRT7/USP39/FOXM1 positive feedback axis.

### Supplementary Information

**Additional file 1: Figure S1.** SIRT7 knockdown affects apoptosis, autophagy, and ROS accumulation in mouse tumor cells. (A) Flow cytometry was used to assess the apoptosis rate in mouse tumor cells after SIRT7 knockdown. (B) Western blot was used to detect FOXM1, USP39, SIRT7 and the key proteins related to autophagy (LC3-I, LC3-II) in mouse tumors after transfection of shNC and shSIRT7#1. (C) DCFH-DA staining was used to detect the ROS levels in mouse tumor cells after shNC and shSIRT7#1 transfection. All results are representative of at least 3-independent experiments. ***p<0.001. **Figure S2.** The network of mechanisms of the SIRT7/USP39/FOXM1 AXIS in human organizations CSCC tissue. (A) Co-IP assay was conducted to evaluate the interaction between USP39 and SIRT7 in human CSCC tissue. (B) The interaction between FOXM1 and USP39 was explored using RIP assay in human CSCC tissue. (C) ChIP assay was performed to explore the interaction between FOXM1 and SIRT7 in human CSCC tissue. All results are representative of at least 3-independent experiments. **p<0.01, ***p<0.001.

## Data Availability

The datasets generated during and/or analyzed during the current study are available from the corresponding author upon reasonable request.
