# Supplementary material for: USP39 interacts with SIRT7 to promote cervical squamous cell carcinoma by modulating autophagy and oxidative stress via FOXM1
Source: J Transl Med. 2023 Nov 13;21:807. doi: 10.1186/s12967-023-04623-4 (PMC10641974; doi:10.1186/s12967-023-04623-4)
Supplement: Supplementary file 1 — Additional file 1: Figure S1. SIRT7 knockdown affects apoptosis, autophagy, and ROS accumulation in mouse tumor cells. (A) Flow cytometry was used to assess the apoptosis rate in mouse tumor cells after SIRT7 knockdown. (B) Western blot was used to detect FOXM1, USP39, SIRT7 and the key proteins related to autophagy (LC3-I, LC3-II) in mouse tumors after transfection of shNC and shSIRT7#1. (C) DCFH-DA staining was used to detect the ROS levels in mouse tumor cells after shNC and shSIRT7#1 transfection. All results are representative of at least 3-independent experiments. ***p<0.001. Figure S2. The network of mechanisms of the SIRT7/USP39/FOXM1 AXIS in human organizations CSCC tissue. (A) Co-IP assay was conducted to evaluate the interaction between USP39 and SIRT7 in human CSCC tissue. (B) The interaction between FOXM1 and USP39 was explored using RIP assay in human CSCC tissue. (C) ChIP assay was performed to explore the interaction between FOXM1 and SIRT7 in human CSCC tissue. All results are representative of at least 3-independent experiments. **p<0.01, ***p<0.001. [file 12967_2023_4623_MOESM1_ESM.docx]

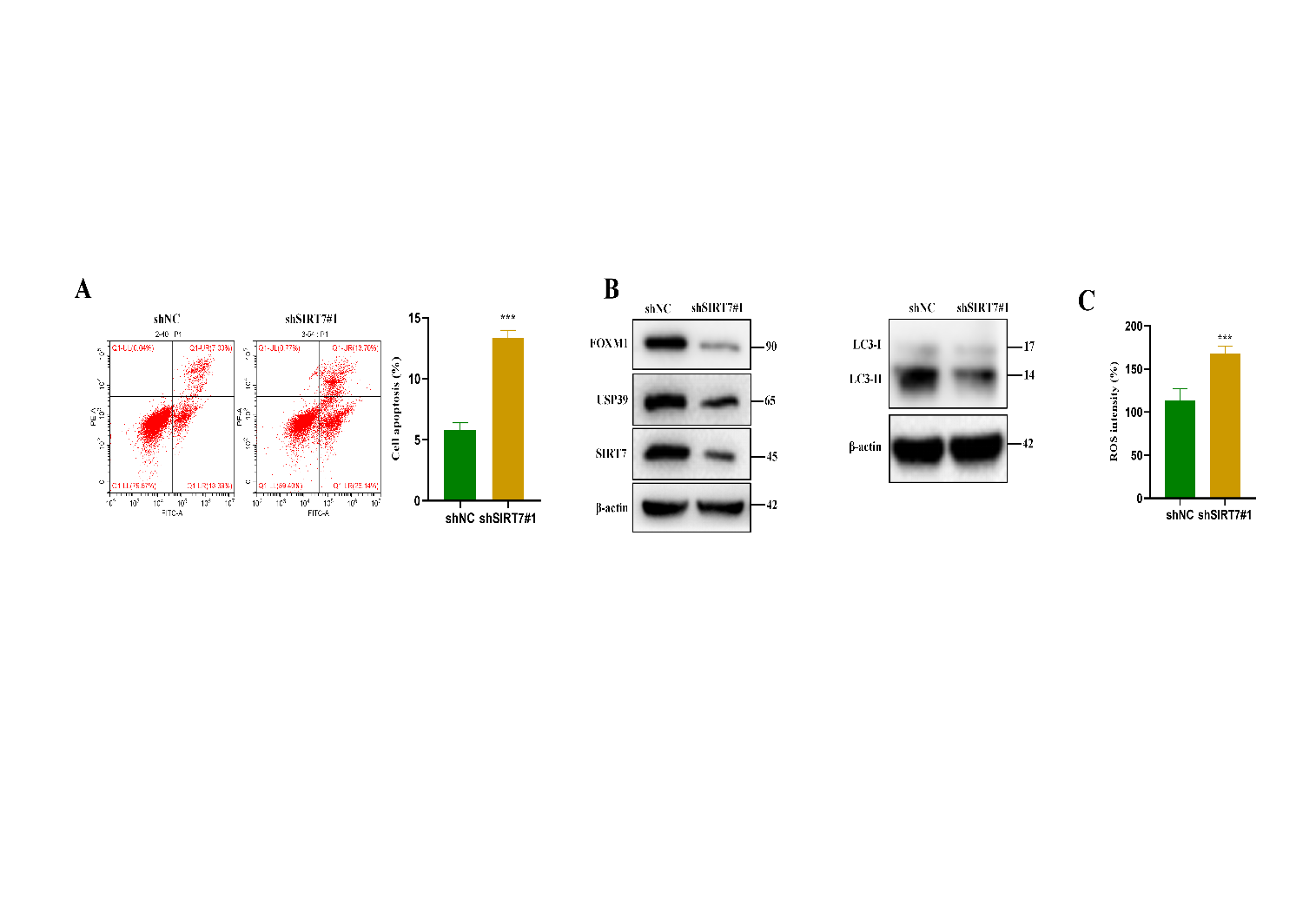


**Figure S1. SIRT7 knockdown affects apoptosis, autophagy, and ROS accumulation in mouse tumor cells.** (A) Flow cytometry was used to assess the apoptosis rate in mouse tumor cells after SIRT7 knockdown. (B) Western blot was used to detect FOXM1, USP39, SIRT7 and the key proteins related to autophagy (LC3-I, LC3-II) in mouse tumors after transfection of shNC and shSIRT7#1. (C) DCFH-DA staining was used to detect the ROS levels in mouse tumor cells after shNC and shSIRT7#1 transfection. All results are representative of at least 3-independent experiments. ***p<0.001.


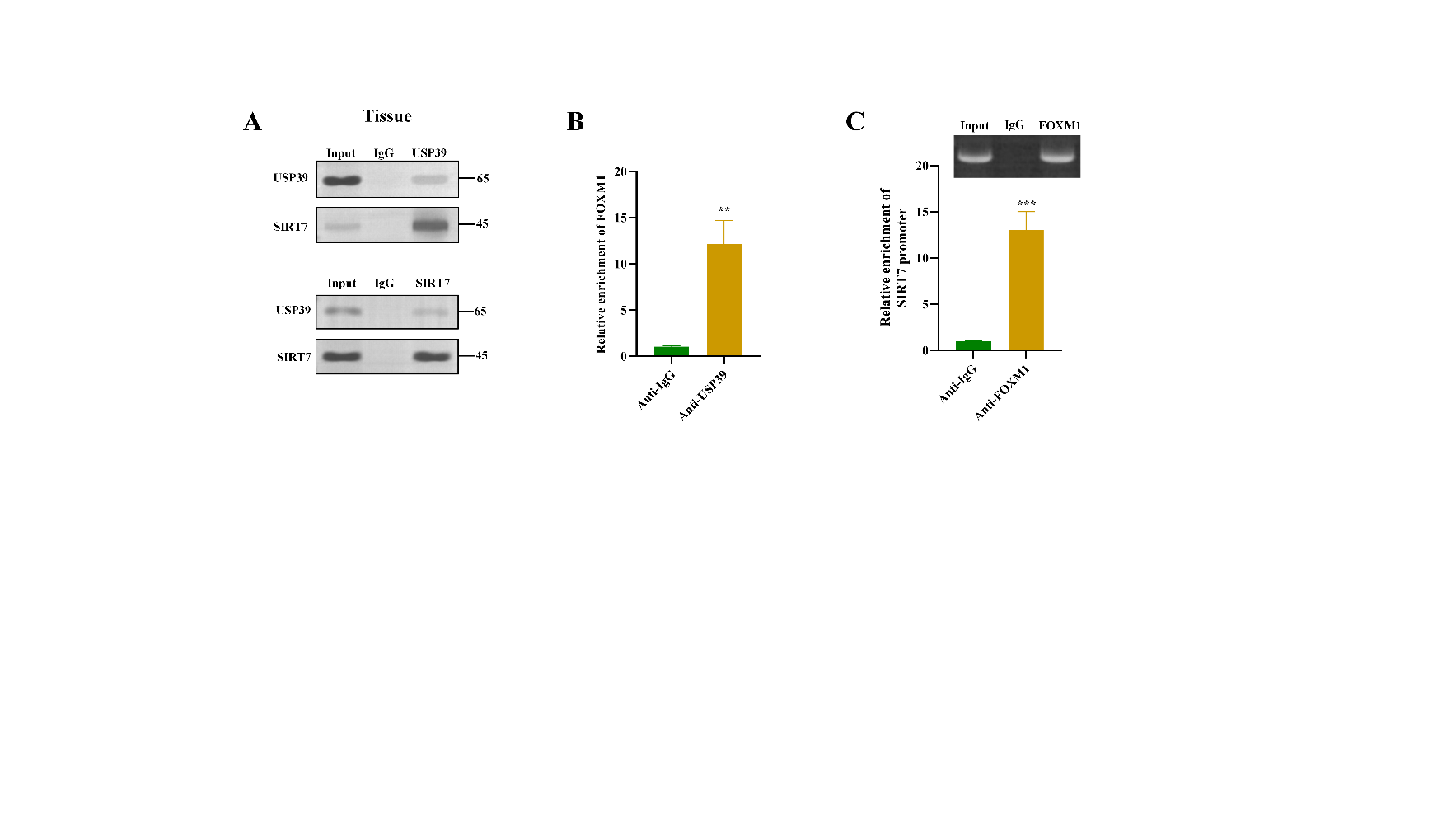


**Figure S2. The network of mechanisms of the SIRT7/USP39/FOXM1 AXIS in human organizations** **CSCC tissue.** (A) Co-IP assay was conducted to evaluate the interaction between USP39 and SIRT7 in human CSCC tissue. (B) The interaction between FOXM1 and USP39 was explored using RIP assay in human CSCC tissue. (C) ChIP assay was performed to explore the interaction between FOXM1 and SIRT7 in human CSCC tissue. All results are representative of at least 3-independent experiments. **p<0.01, ***p<0.001.
